# Antiviral Treatment Reveals a Cooperative Pathogenicity of Baculovirus and Iflavirus in *Spodoptera exigua*, a Lepidopteran Insect

**DOI:** 10.4014/jmb.2012.12045

**Published:** 2021-01-22

**Authors:** Miltan Chandra Roy, Shabbir Ahmed, Md. Mahi Imam Mollah, Yonggyun Kim

**Affiliations:** Department of Plant Medicals, College of Life Sciences, Andong National University, Andong 36729, Republic of Korea

**Keywords:** *Spodoptera exigua*, SeMNPV, iflavirus, dsRNA, viral transmission

## Abstract

NPVThe beet armyworm, *Spodoptera exigua*, is a serious insect pest infesting various vegetable crops. Two infectious insect viruses, baculovirus and iflavirus, are known to induce epizootics in *S. exigua* populations. Indeed, some laboratory colonies have appeared to be covertly infected by these viruses. Diagnostic PCR tests detected two different viruses: *Spodoptera exigua* multiple nucleopolyhedrosis virus (SeMNPV) and iflaviruses (SeIfV1 and SeIfV2). Viral extract from dead larvae of *S. exigua* could infect Sf9 cells and produce occlusion bodies (OBs). Feeding OBs to asymptomatic larvae of *S. exigua* caused significant viral disease. Interestingly, both SeIfV1 and SeIfV2 increased their titers at late larval stages. Sterilization of laid eggs with 1% sodium hypochloride significantly reduced SeMNPV titers and increased larval survival rate. Doublestranded RNA (dsRNA) specific to SeIfV1 or SeIfV2 significantly reduced viral titers and increased larval survival rate. To continuously feed dsRNA, a recombinant *Escherichia coli* HT115 expressing SeIfV1-dsRNA was constructed with an L4440 expression vector. Adding this recombinant *E. coli* to the artificial diet significantly reduced the SeIfV1 titer and increased larval survival. These results indicate that laboratory colony collapse of *S. exigua* is induced by multiple viral infections. In addition, either suppression of SeMNPV or SeIfV infection significantly increased larval survival, suggesting a cooperative pathogenicity between baculovirus and iflavirus against *S. exigua*.

## Introduction

Baculoviruses are known to produce occlusion bodies (OBs) including rod-shaped nucleocapsids (virions). They are divided into nucleopolyhedroviruses (NPVs) and granuloviruses (GVs) [1]. NPVs have multiple virions within each OB, whereas GVs usually contain a single virion in a granular OB form. These OBs are made of genetically related polyhedrin or granulin proteins that are not related to other occlusion proteins of entomopoxviruses or cypoviruses. Each virion of NPVs may envelope single or multiple nucleocapsids (traditionally called SNPV or MNPV, respectively). However, these baculoviruses are classified based on the order of their host insects, including alphabaculoviruses (lepidopteran NPVs), betabaculoviruses (lepidopteran GVs), gammabaculoviruses (hymenopteran NPVs), and deltabaculoviruses (dipteran NPVs) [2]. Alphabaculoviruses are divided into Group I and Group II depending on the nature of a fusion protein (GP64 for Group I versus F protein for Group II) required for the entry of enveloped viruses into host cells [3].

Baculovirus genomes are double-stranded, circular, supercoiled DNAs with sizes ranging from 82 kb (*Neodiprion lecontei* NPV) to 179 kb (*Xestia c-nigrum* GV) [4]. Despite such genome size variation, only 31 genes appear to be common to all baculoviruses, including viral structural proteins, DNA polymerase, transcriptional factors, and alpha-amanitin-resistant RNA polymerase (Rohrmann, 2008). The large number of non-overlapping genes suggests that baculoviruses have co-evolved with their hosts and diverged genetically during evolution [5].

Baculoviruses produce two structurally distinct forms of virus particle: budded virus (BV) and occlusion-derived virus (ODV). BV and ODV are identical in their genomes. Although they have similar nucleocapsids, they have different envelope proteins and lipid contents. BV is produced when nucleocapsids bud off from infected cells. It then acquires a lipoprotein envelope containing a virus-encoded fusion protein of GP64 or F. In contrast, ODV is produced in the nucleus of infected cells. It contains specific envelope components including five per os infectivity factors [6].

The beet armyworm, *Spodoptera exigua* (Hübner) (Lepidoptera: *Noctuidae*), is highly polyphagous. It is considered to be one of the most devastating pests, infesting over 80 crop species grown in fields and greenhouses worldwide and having significant economic impacts [7]. Due to rapid development of resistance to multiple agents and ecological effects of excessive dependence on chemical insecticides for controlling this pest [8], NPV has been widely acknowledged as an environmentally friendly alternative to chemical insecticides [9]. NPV-infected larvae always exhibit no feeding behavior, pale body color, and swelling, resulting in death.

*S. exigua* multiple nucleopolyhedrovirus (SeMNPV) has been successfully applied as a large-scale commercial biological insecticide against *S. exigua* [10]. SeMNPV is a Group II alphabaculovirus with a 128 kb dsDNA genome and 127 putative open reading frames (ORFs) forming a nucleocapsid with proteins [11]. One to seven viral particles are enveloped in a polyhedron (~1.4 μm) that exhibits high host specificity. SeMNPV infects the larvae of *S. exigua*, but not those of other closely related lepidopteran species [11].

Iflavirus (IfV) is a group of single-stranded RNA viruses infecting arthropods. These viruses belong to the order Picornavirales [12]. Two iflaviruses (SeIfV1 and SeIfV2) are known in *S. exigua*. They can form icosahedral capsids of 27 nm in diameter. The genome size is 10.3 nucleotides for SeIfV1 and 9.4 nucleotides for SeIfV2. Their genomes have a single ORF to be translated as a single polypeptide that is subsequently cleaved into functional and structural proteins [13, 14]. Although some IfVs can cause clear infection signs (e.g., those infecting *Bombyx mori* [15] and *Apis mellifera* [16]), most IfV infections are asymptomatic [17]. To enhance viral pathogenicity, IfV has been treated with baculoviruses in a mixture [18]. In fact, *S. exigua* collected in the field could be infected with both SeMNPV and two iflaviruses [19]. OBs of SeMNPV produced in insects infected by both viruses contain IfV genomes [20]. Overt infection of SeIfVs can potentiate the viral pathogenicity of SeMNPV [21].

Viruses can cause devastating epizootics of disease in insect colonies. Thus, continuous monitoring and disinfection processes are needed to maintain colony health [22, 23]. IfVs are rapidly transmitted and can reach a high prevalence of infection in natural populations and laboratory insect colonies [24]. Recent laboratory colony collapses in various places in Korea suggest that *S. exigua* colonies might be infected with SeMNPV and SeIfVs. This study confirmed infections by both SeMNPV and SeIfVs in *S. exigua* and showed their cooperative pathogenicity to *S. exigua* using antiviral treatments.

## Materials and Methods

### Rearing *S. exigua*

Larvae of *S. exigua* used in this study originated from a welsh onion (*Allium fistulsum* L.) field in Andong, Korea and were reared for 25 years in a laboratory at 25 ± 2°C with a 16:8 h light/dark cycle and relative humidity of 60 ± 5%. The larvae were fed an artificial diet and underwent five instars (L1-L5) [25]. Adults were supplied with a 10%sucrose solution. Occasionally, field populations were introduced to the laboratory colony to prevent random genetic drift.

### PCR Diagnosis of SeMNPV, SeIfV1, and SeIfV2

For diagnosis of SeMNPV contamination, genomic DNA (gDNA) was extracted from *S. exigua* larvae using 5%Chelex (Bio-Rad, USA). For diagnosis of SeIfVs, RNA samples were extracted from the whole body or different tissues (hemocytes, fat body, midgut, and epidermis) of L5 larvae of *S. exigua* using Trizol reagent (Invitrogen, USA) according to the manufacturer’s instructions. Extracted RNA was resuspended in nuclease-free water and quantified using a spectrophotometer (NanoDrop, Thermo Scientific, USA). RNA (1 μg) was used for cDNA synthesis with an RT PreMix (Intron Biotechnology, Korea) containing oligo dT primer according to the manufacturer’s instructions. PCR was conducted using DNA Taq polymerase (GeneALL, Korea) with gene-specific primers (Table S1). After heat treatment at 94°C for 5 min, PCR was performed with 40 temperature cycles of 94°C for 1 min, 52°C for 1 min, and 72°C for 1 min. A ribosomal protein, *RL32*, was used as a reference gene to confirm cDNA preparation.

### Quantitative PCR (qPCR) to Monitor SeMNPV, SeIfV1, and SeIfV2 Titers During *S. exigua* Development

qPCR was performed to quantify mRNAs of IfV and SeMNPV using a real-time PCR machine (StepOnePlus Real-Time PCR System, Applied Biosystems, Singapore) with Power SYBR Green PCR Master Mix (Life Technologies, USA) according to the guidelines of Bustin *et al*. [26]. For SeIfV1 and SeIfV2, RNA samples were extracted from different developmental stages (100 eggs, 20 young larvae (L1-L3), three L4 larvae, one L5 larva, one pupa, and one adult) using Trizol reagent. Extracted RNAs were dissolved in 50 μl of diethyl pyrocarbonate-treated deionized and distilled water. Their concentrations were quantified with a spectrophotometer. cDNAs were synthesized using 1 μg of total RNA and Maxime RT PreMix (Intron Biotechnology). Synthesized cDNAs were used as templates for qPCR amplification. The reaction mixture (20 μl) contained 10 μl of Power SYBR Green PCR Master Mix, 3 μl of cDNA template (50 ng), 1 μl each of forward and reverse primers (Table S1), and 5 μl of sterile water. In case of SeMNPV, gDNA (50 ng) was used as template. qPCR cycling began with heat treatment at 95°C for 10 min followed by 40 cycles of denaturation at 94°C for 30 s, annealing at 52°C for 30 s, and extension at 72°C for 30 s. The expression level of a ribosomal protein gene, *RL32*, was used as a reference to normalize target gene expression levels after different treatments. PCR specificity was assessed by melting curve analysis. Quantitative analysis was performed using the comparative CT (2^-ΔΔCT^) method [27].

### SeMNPV Isolation and Infection of Sf9 Cells

Baculovirus was isolated from infected larvae followed the method of Choi *et al*. [28]. Briefly, 25 infected L5 larvae of *S. exigua* were taken into a 50 ml tube containing 10 ml sterilized water and macerated using a sterilized pestle. The sample was then filtered through five layers of cheese cloth before being centrifuged at 4,000 ×*g* for 5 min. The supernatant was transferred to a new tube, filtered through a 0.22 μm filter (Acrodisc, Pall Corporation, USA), and used for subsequent bioassay. The Sf9 cell line (IPLB-Sf21-AE) was derived from *Spodoptera frugiperda* pupal ovarian tissue. Sf9 cells were infected with the filtrate at a ratio of 1 ml of filtrate in 5 ml of confluent culture. After 24 h of incubation at 28°C, the culture medium was replaced with fresh TC-100 cell culture medium (Welgene, Korea) containing 5% fetal bovine serum (FBS, Welgene) and cultured for another 48 h. Then the media containing cells were centrifuged at 1,000 ×*g* for 3 min. The supernatant was collected into a 15 ml fresh tube and used for bioassay subsequently.

### Plaque Assay to Quantify SeMNPV

Viral titer was determined by plaque assays. Briefly, one day before the assay, Sf9 cells were plated in 6-well plates at a density of 80%. The virus was serially diluted up to 10^-5^ to 10^-10^ with the cell culture medium. Cells were infected with 200 μl of each virus dilution by adding it to the culture medium and evenly distributed it over the monolayer of cells. The virus was allowed to infect the cells by incubation at 37°C for 60 min. During this time, an agarose overlay medium was prepared by melting 5 ml of 5% agarose and cooling down to 44°C. Then 50 ml of a warm (44°C) cell culture medium was added to the agarose. The virus-containing medium was removed from cells and replaced with the agarose solution. Plaques were observed after 7 days of incubation at 28°C. Observation of plaques was done by adding 1 ml of neutral red solution (0.03% in PBS) to each well followed by incubation at 37°C for 3 h.

### RNA interference (RNAi) against SeIfV1 and SeIfV2

RNAi was performed using double-stranded RNAs (dsRNAs) specific to SeIfV1 and SeIfV2, respectively. These dsRNAs were prepared using a MEGAscript RNAi Kit (Ambion, USA) according to the manufacturer’s instructions. DNA fragments were obtained by PCR using virus-specific primers (Table S1) containing T7 promoter sequence at the 5' end. Sense and antisense RNA strands were synthesized using T7 RNA polymerase at 37°C for 4 h. The resulting dsRNA was mixed with transfection reagent Metafectene PRO (Biontex, Germany) at 1:1 (v/v) ratio and then incubated at 25°C for 30 min to form liposomes. Then, 1 μg of dsRNA was injected into the hemocoel of apparently healthy L5 larvae using a microsyringe (Hamilton, USA) equipped with a 26-gauge needle. At 12 h post injection, RNAi efficacy was determined by reverse transcription (RT)-qPCR as described above. A green fluorescence protein (*GFP*) gene was used as a control dsRNA (dsCON) [29]. Each treatment was replicated three times using independent RNA preparations.

### Construction of Recombinant *Escherichia coli* Expressing dsRNA Specific to SeIfV1

To clone a partial genomic fraction (188 bp) of SeIfV1, L4440 plasmid containing multiple cloning sites flanked by two T7 promoters in an inverted orientation was used. Restriction sites of HindIII and XbaI were chosen for cloning because these sites were not present in SeIfV1 based on analysis using the Gene-Quest program (DNASTAR, USA). This recombinant L4440 vector containing SeIfV1 was then transformed into *E. coli* HT115 (DE3) competent cells lacking RNase III by electroporation.

### Overexpression of dsRNA in Transformed *E. coli* and Quantification

To produce dsRNA, a single colony of transformed bacteria was cultured in Luria-Bertani (LB) containing 100 μg/ml ampicillin at 37°C with shaking at 250 rpm for 16 h. The culture broth (5 ml) was added to 500 ml of fresh LB medium containing 100 μg/ml ampicillin and allowed to grow at 37°C until it reached an exponential bacterial growth phase with absorbance of 0.6~0.7 at 600 nm. Expression of T7 polymerase was induced by adding 0.4 mM (final concentration) of isopropyl thiogalactose (IPTG) and then further cultured at 30°C for 6 h. Total bacterial RNA was extracted with an RNA Extraction Mini Kit (Qiagen Korea, Seoul, Korea). The dsRNA was confirmed by 1% agarose gel electrophoresis. For bioassays, IPTG-induced recombinant bacterial culture was centrifuged at 7,000 ×*g* for 10 min. The resulting bacterial cell pellet was resuspended in distilled water. The bacterial suspension was then treated at 95°C for 10 min. Subsequently, the bacterial suspension was subjected to ultrasonication at 100% intensity for 3 cycles (5 second per cycle) with an ultrasonicator (Bandelin Sonoplus, Germany). Quantification of dsRNA amounts produced by recombinant bacteria followed the method described by Kim *et al*. [30].

### Feeding Bioassay Using dsRNA-Expressing Recombinant Bacteria

Recombinant bacteria (10 μl, 7 × 10^9^ cells/ml) were mixed with 50 mg of larval artificial diet. This treated diet was used to feed 30 larvae at early L1. Every 48 h, the diet was replaced with a freshly treated diet. As a control, non-recombinant HT115 bacteria were used to treat the diet. Each treatment was replicated three times. For each replication, 30 larvae were used. Survival rates were estimated for each developmental stage from L1 larvae to adults.

### Egg Sterilization Using Sodium Hypochlorite (NaOCl)

NaOCl solution was purchased from Yakuri Pure Chemicals (Japan). Different concentrations (0.1%, 0.5%, and 1%) of NaOCl were used to treat *S. exigua* eggs by diluting a stock solution of 12% NaOCl with sterilized deionized water. Eggs were incubated in NaOCl for 5 min and then rinsed with sterile water for 2 min. Treated eggs were dried for 30 min on a clean bench. To examine the effect of immersion time on SeMNPV titer and survival, eggs were also treated with 1% NaOCl for 5 min, 10 min, and 20 min followed by a 2 min water rinse. Each treatment was replicated three times (30 eggs for each replication).

### Statistical Analysis

Data from all assays were analyzed by one-way Analysis of Variance (ANOVA) of PROC GLM for continuous variables using SAS program [31]. All studies were plotted by mean ± standard error using Sigma plot. Means were compared by a least squared difference (LSD) and discriminated at Type I error = 0.05. The significance of difference between the two groups was analyzed using Student’s *t*-test of Sigma plot software 12.0 version. *p*-value < 0.05 was considered to be statistically significant for Student’s t-test.

## Results

### Colony Collapse with Viral Infection Symptoms

**Fig. 1:**
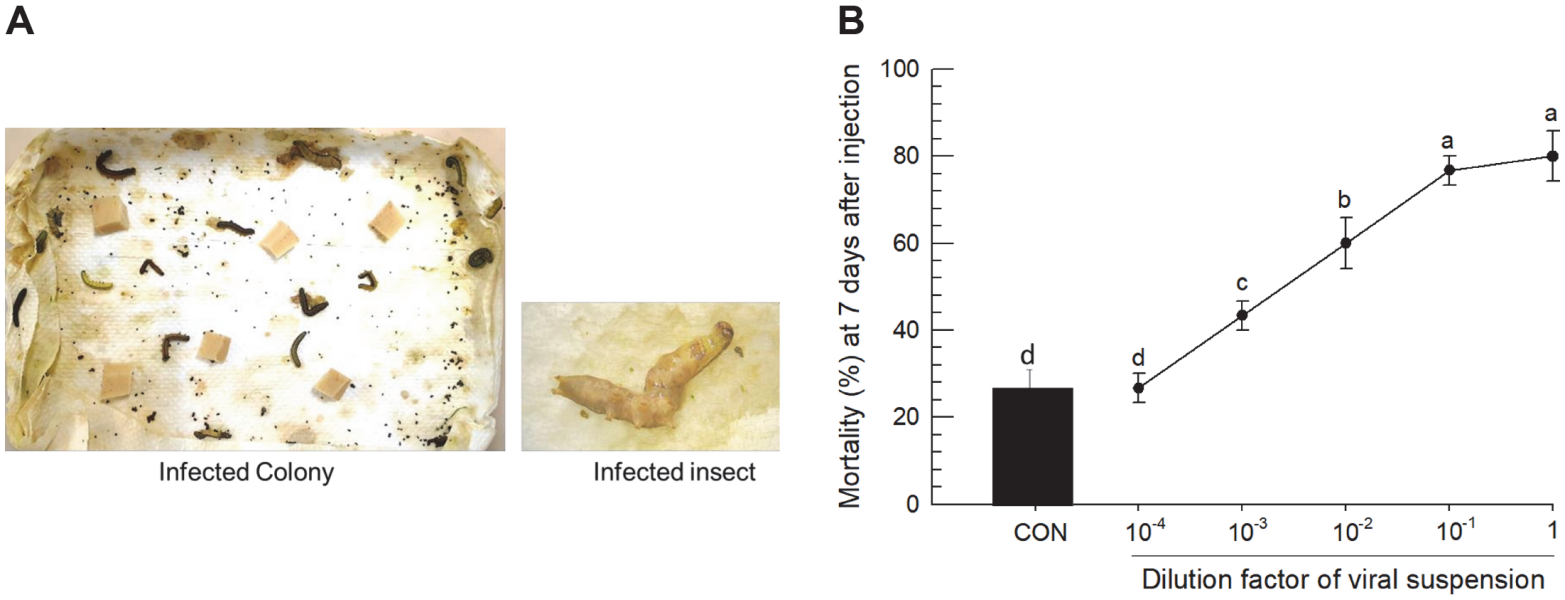
Colony collapse of *S. exigua* with viral symptoms. (**A**) Colony collapse symptom of *S. exigua* larvae showing flacherie symptoms. (**B**) Mortality of *S. exigua* larvae at 7 days after injection of SeMNPV viral extract. Viral particles were extracted from 25 infected fifth instar larvae and 2 μl of the extract was injected into healthy L5 larvae. Each dose was replicated three times (ten larvae for each replication). Different letters above standard error bars indicate significant difference among means of each species at Type I error = 0.05 (LSD test).

A laboratory colony has been maintained for the last 25 years with an artificial diet described in Materials and Methods. Its population size varied depending on the handling techniques of researchers. Recently, the laboratory population suddenly died, mostly at the late larval stage, with virus infection-like symptoms (Fig. 1A). The dead larval bodies were melted (wilted) and had released hemolymph. These larvae were collected and used to isolate viral particles by filtration and centrifugation. The pellet was resuspended in sterilized water and used for injection to non-symptomatic larvae. Treated larvae exhibited the same viral infection-like symptoms. The viral extract killed the larvae in a dose-dependent manner depending on the dilution factor (Fig. 1B).

### Detection of Viral Pathogens Using PCR Diagnosis

**Fig. 2:**
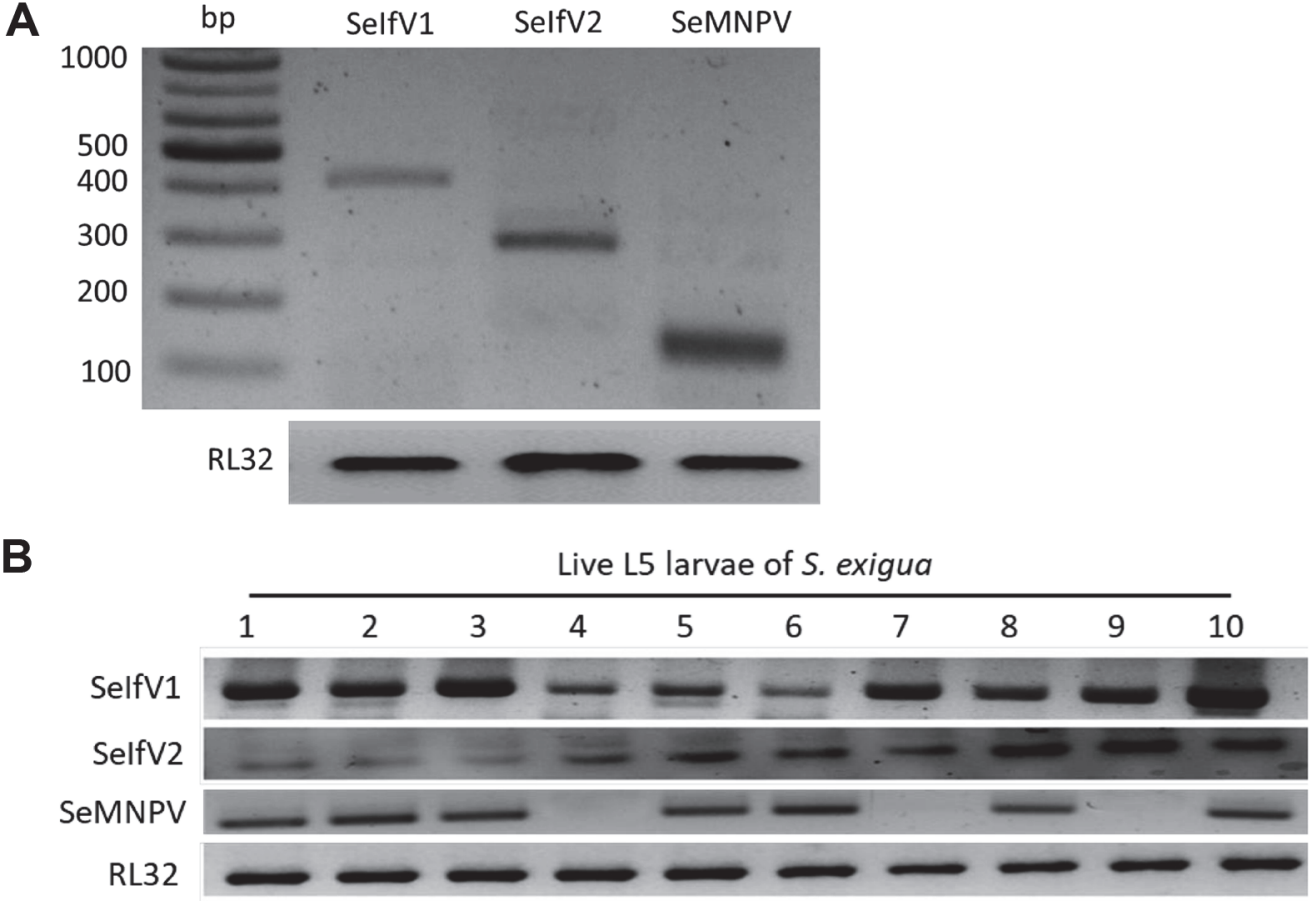
Detection of viral pathogens using PCR diagnosis. (**A**) SeMNPV and SeIfVs were detected using gene-specific primers (Table S1). (**B**) Infectivity assessment of SeMNPV and SeIfVs using randomly selected ten L5 larvae of a laboratory colony. A ribosomal protein, *RL32*, was used to validate cDNA integrity. PCR products were separated on agarose (1%) gel.

In other laboratory colonies, *S. exigua* has been reported to show covert infection with baculovirus and iflaviruses [32]. We tested our laboratory colony with three diagnostic primers specific for SeMNPV and two IfV species. Results of PCR diagnosis showed that our laboratory colony was infected with SeMNPV, SeIfV1, and SeIfV2 (Fig. 2A). Since L5 larvae showed most viral infection symptoms, they were randomly selected for PCR diagnosis (Fig. 2B). All larvae were infected by two SeIfV viruses, while not all larvae were infected by SeMNPV.

### SeMNPV Isolation and Infectivity

**Fig. 3:**
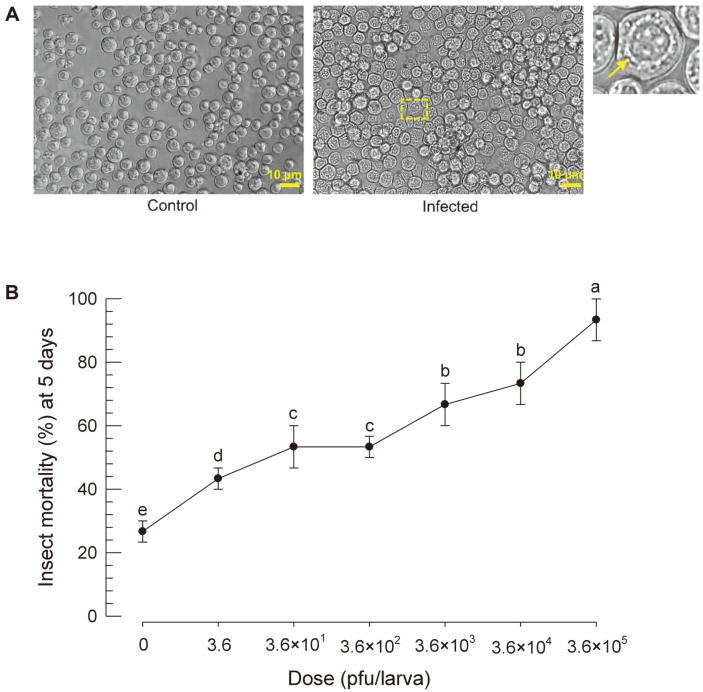
Polyhedral formation of a viral isolate in Sf9 cells and its infectivity in *S. exigua*. (**A**) Polyhedra (see a magnified square) in Sf9 cells infected with a viral isolation from larvae exhibiting a virus infection-like symptom. In contrast, non-infected Sf9 cells did not show any polyhedral in their cytoplasm. (**B**) Pathogenicity of budded viruses (BVs) quantified with plaque assays. BV suspension was injected into L5 larvae (1 μl per larva). Mortality was assessed at 24 h after injection and daily thereafter for up to 5 days. Each BV dose was replicated three times (ten larvae for each replication). Different letters above standard error bars indicate significant difference among means of each species at Type I error = 0.05 (LSD test).

To observe the OB particles of SeMNPV, Sf9 cells were infected with viral extract (Fig. 3A). At 3 days post-infection, infected Sf9 cells formed viral particles. The cultured medium presumably containing the BV form of the SeMNPV was quantified with a plaque assay and injected into larvae (Fig. 3B). SeMNPV caused mortality of *S. exigua* in a dose-dependent manner.

### Viral Replication of SeIfV1 and SeIfV2

**Fig. 4:**
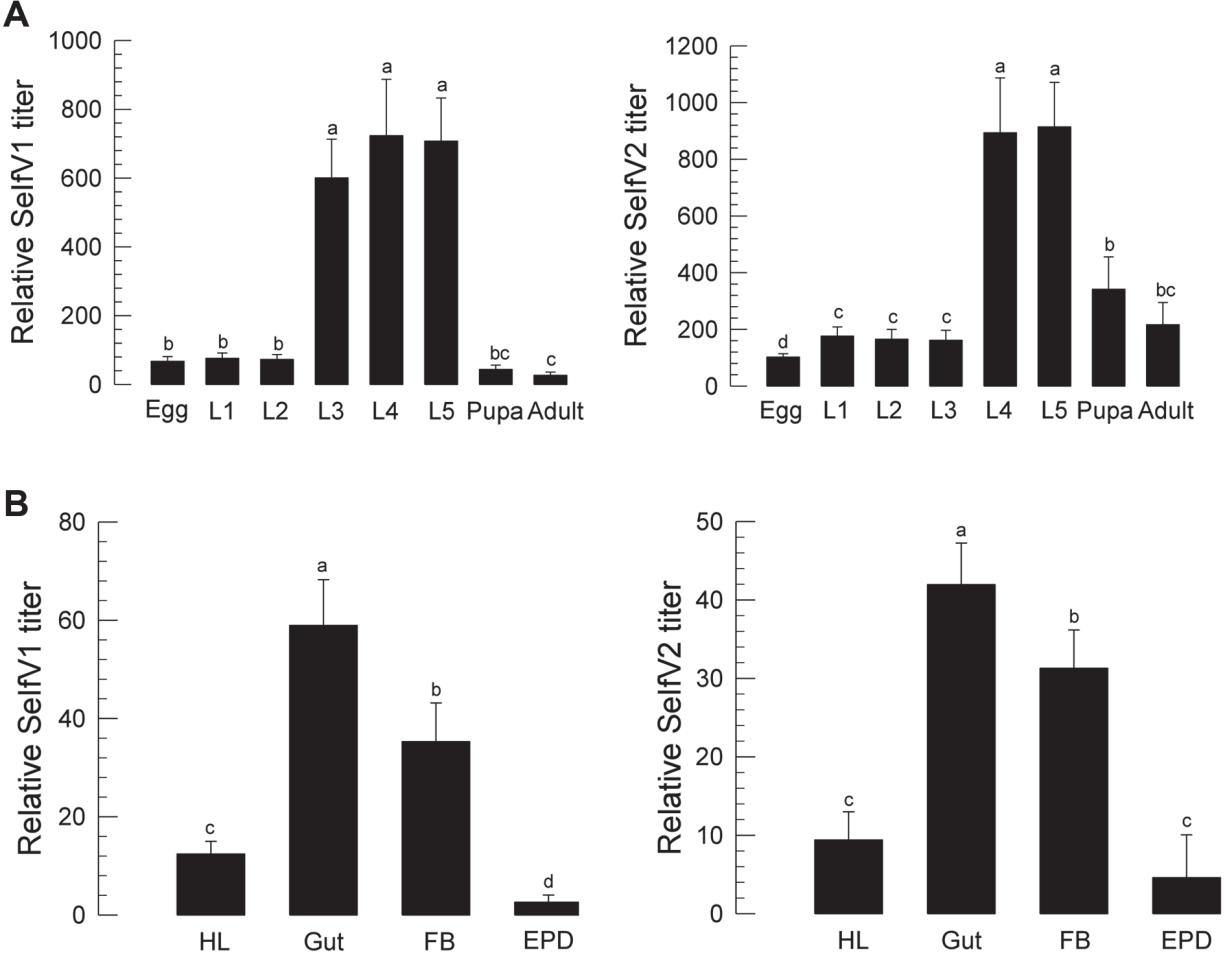
Viral replication of SeIfV1 and SeIfV2 in different developmental stages and tissues of *S. exigua*. Viral titers of SeIfV1 and SeIfV2 were quantified by RT-qPCR. (**A**) Different developmental stages of egg, 1st to 4^th^ instar (‘L1‐L4’), pupa, and adult. (**B**) Different tissues in L5 larvae: midgut (‘Gut’), fat body (‘FB’), epidermis (‘EPD’), and hemolymph (‘HL’). Different letters above standard error bars indicate a significant difference among means at Type I error = 0.05 (LSD test).

Two iflaviruses were relatively titrated in the laboratory population of *S. exigua* with qPCR (Fig. 4). Both IfVs were detected in all developmental stages, with late larval stages exhibiting relatively high viral levels (L3-L5 stages for SeIfV1 and L4-L5 stages for SeIfV2) (Fig. 4A) presumably due to viral replication. In L5 larvae, both IfVs were highly detected in the midgut and fat body (Fig. 4B).

### Egg Sterilization and SeMNPV Viral Titers

**Fig. 5:**
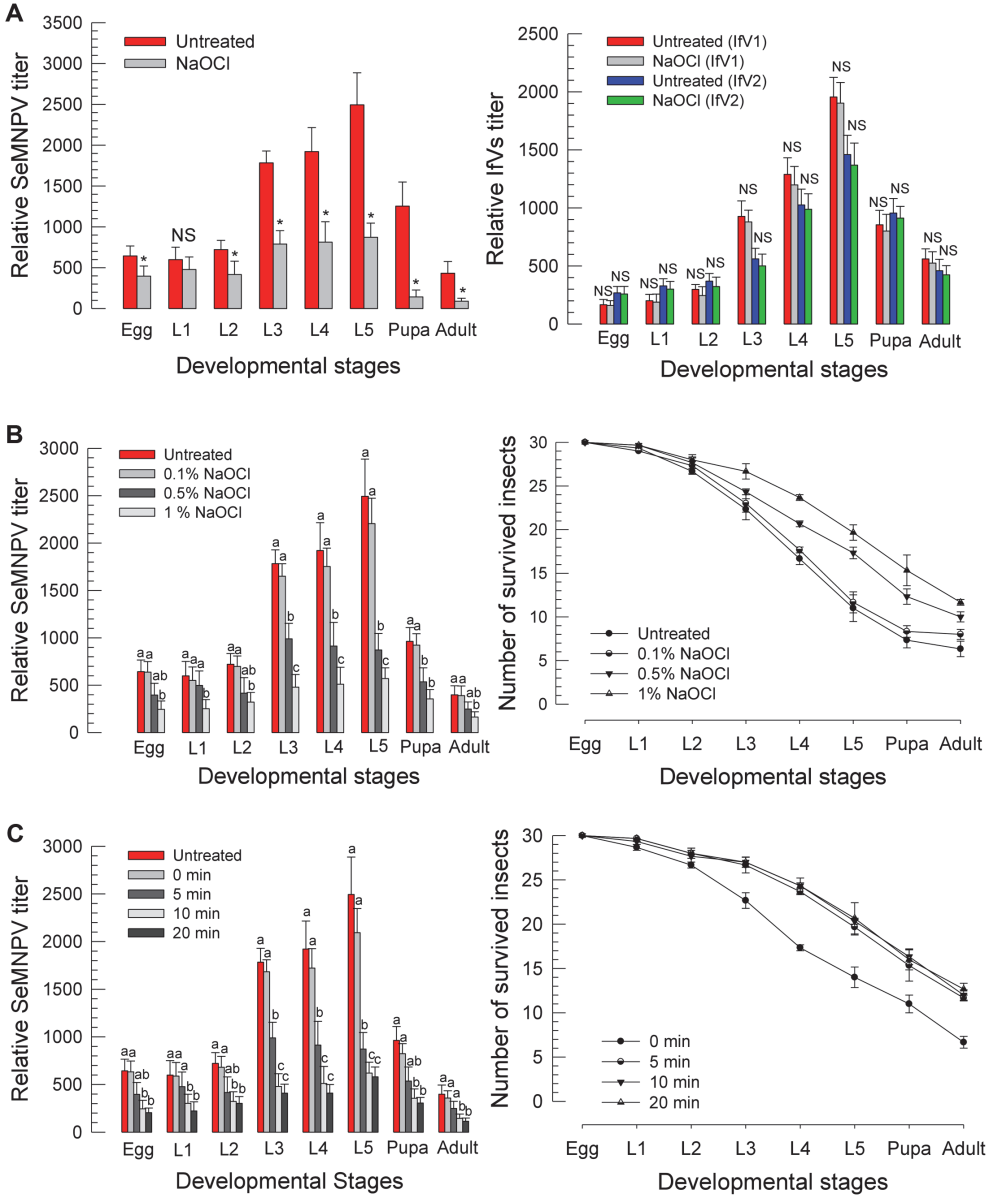
Effects of egg sterilization using NaOCl on SeMNPV and SeIfV titers in different developmental stages of *S. exigua*. (**A**) RT-qPCR quantification of SeMNPV and SeIfV titers. Eggs were sterilized with 0.5% NaOCl for five minutes. (**B**) RT-qPCR quantification of SeMNPV titers. Eggs were sterilized with 0.1, 0.5, and 1% NaOCl for 5 min. (**C**) Quantification of SeMNPV titers after sterilizing eggs with 1% NaOCl for 0, 5, 10, 20 min and the survivorship of *S. exigua*. Different letters above standard error bars indicate a significant difference among means at Type I error = 0.05 (LSD test).

Sodium hypochloride (NaOCl) was used to sterilize viral contamination [33]. Egg sterilization with NaOCl suppressed SeMNPV viral titers in all developmental stages (Fig. 5A). However, NaOCl treatment did not suppress IfV titers. An increase of NaOCl concentration was effective in suppressing SeMNPV level and increasing larval survival rate (Fig. 5B). Five minutes of incubation time with 1% NaOCl was optimal for SeMNPV sterilization (Fig. 5C).

### RNAi Treatment against SeIfV1 and SeIfV2

**Fig. 6:**
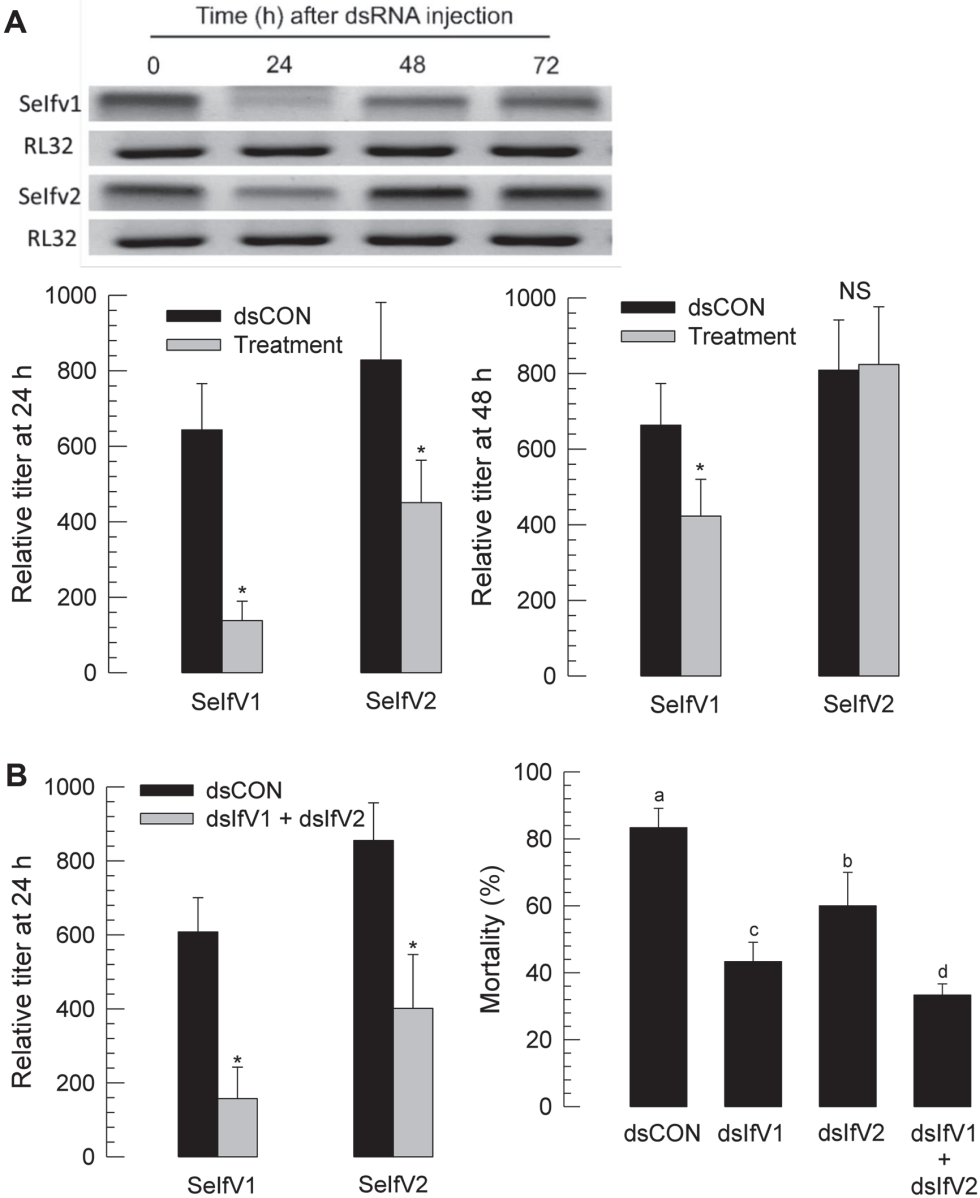
RNAi treatment against SeIfV1 and SeIfV2. (**A**) RNAi efficiency was assessed by RT-qPCR. *RL32* was used as a reference gene of RT-qPCR to normalize target gene expression level. Each treatment was replicated with independently prepared samples. (**B**) Effects of RNAi specific for SeIfV1 and SeIfV2 on survivability. Different letters above standard deviation bars indicate significant difference among means at Type I error = 0.05 (LSD test).

To suppress IfV titers, dsRNA was generated using an in vitro transcription kit. These specific dsRNAs were effective in suppressing SeIfV1 levels for 48 h post-injection (Fig. 6A). However, the SeIfV2 levels were suppressed for only for 24 h and returned to control level at 48 h. The dsRNA treatment significantly (*p*-value < 0.05) rescued larvae from the viral disease (Fig. 6B). Interestingly, treatment with the mixture was highly effective in rescuing larvae from viral disease.

**Fig. 7:**
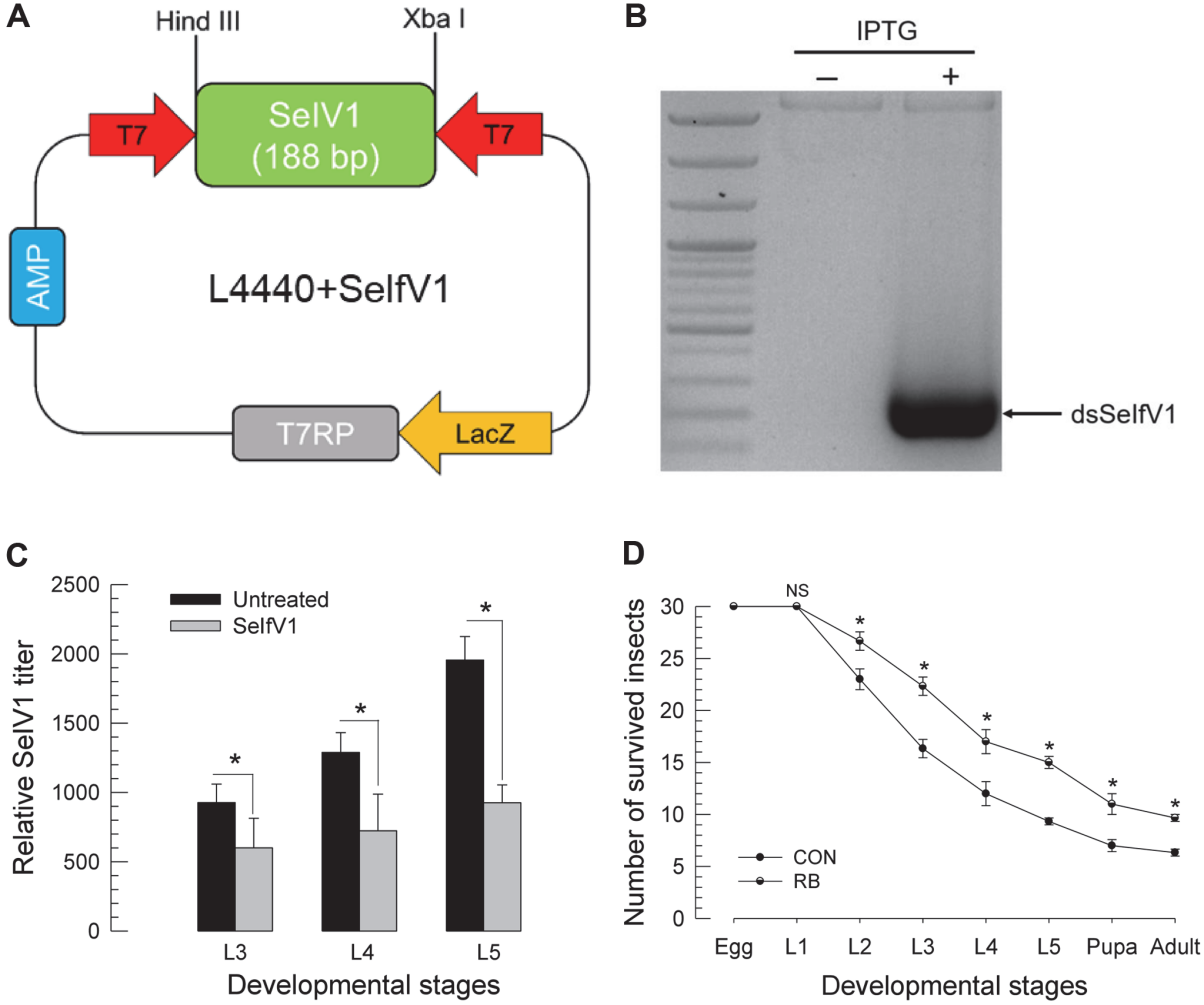
Construction of recombinant *E. coli* expressing dsRNA specific to SeIfV1. (**A**) Cloning of a fragment of SeIfV1 (188 bp) into an expression vector of L4440 using HindIII and XbaI multiple cloning sites. After selecting transformed cells using ampicillin (‘AMP’) medium, T7 RNA polymerase (‘T7 RP’) was overexpressed by IPTG inducer on lactose promoter (‘LacZ’). (**B**) Partial SeIfV1 RNA was transcribed by two opposite T7 promoters (‘T7’) and dsSeIfV1 production specifically in recombinant *E. coli* under IPTG induction was confirmed. (**C**) Effect of recombinant bacteria on SeIfV1 titer. Amounts of SeIfV1 in L3, L4, and L5 stages were determined by qPCR. (**D**) Effect of recombinant bacteria (RB) on survivability. Asterisk (*) above standard deviation bars indicate significant difference among means at Type I error = 0.05 (LSD test).

Construction of Recombinant *E. coli* Expressing dsRNA Specific to SeIfV1 and its Administration to Larvae Oral administration of dsRNA was effective in suppressing target genes in *S. exigua* [30]. To persistently suppress IfV levels, a recombinant *E. coli* expressing dsRNA specific to SeIfV1 was constructed (Fig. 7A). Addition of IPTG inducer to the bacterial culture markedly enhanced dsRNA (Fig. 7B). Induced *E. coli* was harvested and their bacterial cell walls were destabilized by ultrasonication to facilitate dsRNA release after ingestion of bacteria [30]. An artificial diet containing 1% recombinant bacteria was fed to larvae from hatch to pupation (Fig. 7C). The RNA level of SeIfV1 was highly suppressed at larval, pupal, and adult stages. Larvae fed with the recombinant *E. coli* showed significantly increased pupation rate (Fig. 7D).

## Discussion

Insects are attacked by a wide range of viruses. Covert infections are increasingly shown to be common in field populations of Lepidoptera [34]. Here, we reported a laboratory colony collapse of *S. exigua* due to multiple viral infections leading to an epizootic. A colony of *S. exigua* had been maintained in a laboratory for over 25 years but recently exhibited viral infection symptoms. It was found that these larvae were infected by SeMNPV and SeIfV based on PCR diagnosis using the technique developed by Virto *et al*. [32]. Indeed, the Spanish group reported that 54% of the field population are infected by SeMNPV, among which 8.4% of individuals are also infected by SeIfV [32]. In contrast, almost all larvae in our laboratory population were infected by both viral species. Such covert infection with viruses might have been caused by occasional introduction of field populations to maintain the laboratory population. Quantification of SeMNPV viral titers in asymptomatic individuals over two laboratory generations showed a wide range (from 0.29 pg per larva to 3.5 × 10^5^ pg per larva), which resulted in a differential susceptibility to SeMNPV superinfections [35]. These results suggest transmission patterns of baculovirus in the environment and the role of covert infections in host-pathogen interaction dynamics. In Spain, two SeIfV strains (SeIfV1 and SeIfV2) have been reported in wild populations and laboratory colonies of *S. exigua* [36].

Covert infection of SeMNPV is not uncommon. It usually causes spontaneous disease outbreaks. SeMNPV exhibits an oral infection via the peritrophic matrix (PM) and causes midgut cells to invade the hemocoel of *S. exigua* [3, 37]. The insects defend against the viral infection and replication with their potent immune system [38, 39]. PM serves as the frontier physical barrier to deter viral infection of the midgut epithelium [40]. This explains the role of viral metalloprotease called ‘enhancin’ in degrading PM to enhance the pathogenicity of NPV [41]. In addition to PM, the insect midgut also plays a crucial role in fighting against the viral infection by expressing innate immune responses and damage repair machinery [42]. RNA interference, apoptosis, autophagy, and immune signal pathways such as JNK, JAK-STAT, and p38 MAPK are important insect defense machineries against viral infection [43, 44]. Furthermore, several hydrolytic enzymes such as lipase, serine protease, and alkaline trypsin from the midgut of *Bombyx mori* have been found to exhibit remarkable antiviral activities [45-47]. Upon SeMNPV infection, 124 putative innate immunity-related genes are differentially expressed in the larval midgut of *S. exigua*. They are divided into several groups, including pattern recognition proteins, signaling pathways, signal modulation, antimicrobial peptides and detoxification [48].

Two SeIfV strains were detected in the laboratory colony *S. exigua* in the present study. These SeIfVs were pathologically associated with viral diseases because their specific RNAi treatment significantly rescued infected larvae. Iflaviruses can form non-enveloped, icosahedral particles containing a positive single-stranded RNA genome encoding one large polypeptide which is then post-translationally processed into structural viral coat proteins and nonstructural proteins associated with viral replication and protein processing [17]. IfV was initially found to be able to infect insects of economic importance such as honey bees and silkworms, causing diarrhea, developmental malformation, and death along with asymptomatic infection. Sacbrood disease is an example of a fatal IfV infection that prevents larva-to-pupal metamorphosis due to the Sacbrood virus (SBV) which fills in the exuvial space between larval and pupal cuticles in the pharate stage [49, 50]. Deformed wing virus (DWV) is another example of a honey bee virus with symptomless infection. It can induce morphological abnormality in emerging adults, leading to crippled bees [51]. Colony collapse disease (CCD) was reported in honey bees in 2006. CCD bees are known to contain RNA viruses including DWV [52]. In addition, a flacherie symptom exhibiting diarrhea was observed in silkworms. The causative agent was IfV whose genome (9,650 nucleotides) was first sequenced [53]. There appears to be a positive virus-virus interaction between SeIfV and SeMNPV because IfV co-inoculation can consistently enhance the virulence of SeMNPV compared to larvae infected with SeMNPV alone [36]. An electron microscopy analysis revealed the presence of IfV virions inside SeMNPV OBs, suggesting the possible co-occlusion of both viruses [20]. Thus, physical protection such as resistance to UV radiation and high temperature may be offered by SeMNPV OBs, which could increase the infectivity of the IfV. This cooperative pathogenicity was observed in our current study. dsRNA treatment(s) specific to SeIfV1 or SeIfV2 effectively suppressed the IfV titers as shown in Fig. 6. Accordingly, the pathogenicity of the baculovirus was diminished. This decrease of mortality can be interpreted as iflavirus virulence or cooperative pathogenicity of iflavirsus with the baculovirus. However, our study demonstrated this cooperative pathogenicity of IfV with baculovirus because NaOCl treatment decreased SeMNPV titers without any change of IfV titers. This supports the enhanced pathogenicity of baculovirus due to IfV observed in other studies [19, 21]. The observation of IfV in the envelop of baculovirus in an OB form [19] suggests a role of physical protection of IfV by baculovirus. This viral interaction may maintain their co-existence in *S. exigua* populations as observed in this current study.

Egg sterilization with NaOCl was effective in reducing SeMNPV, but not SeIfVs. Effective baculoviral epizootics occur via vertical and horizontal transmissions. These viral transmissions are possible because baculoviral OBs have been shown to persist for considerable periods of time outside their hosts if they are protected from UV such as by being in soil [54]. The sterilization in the current study might have removed external viral contamination of eggs through transovum transmission. However, it cannot completely eliminate the virus carried inside the egg via transovarial transmission [55]. Prevalence of covert infection plays a crucial role in maintaining viral persistence for controlling pest populations under their economic threshold levels in field crops [56]. Due to interest in using insect viruses as potential microbial control agents, baculoviruses that can kill their hosts relatively rapidly have attracted the greatest attention. On the other hand, it is advantageous for baculoviruses to keep their hosts alive and growing as long as possible because there is a negative correlation between the speed-to-kill and the quantity of virus progeny produce. SeIfV did not change the speed-to-kill, although it enhanced SeMNPV virulence, suggesting that they might be an ideal viral complex to control *S. exigua* in field conditions to induce viral epizootics.

Altogether, this study reports multiple viral infections by SeMNPV and SeIfV in a laboratory colony of *S. exigua*. These viral infections resulted in colony collapse of *S. exigua*. Surface sterilization of eggs partially prevented a vertical transmission of SeMNPV. RNAi suppressed SeIfV infection. This study also suggests that these two viruses are cooperative in pathogenicity and that their combined viral infection could be an ideal control tactic against *S. exigua* infesting field crops by inducing a serious viral epizootic.

## Supplemental Materials



Supplementary data for this paper are available on-line only at http://jmb.or.kr.
