# Supplementary material for: Antiviral Treatment Reveals a Cooperative Pathogenicity of Baculovirus and Iflavirus in Spodoptera exigua, a Lepidopteran Insect
Source: J Microbiol Biotechnol. 2021 Jan 22;31(4):529–39. doi: 10.4014/jmb.2012.12045 (PMC9723280; doi:10.4014/jmb.2012.12045)
Supplement: Supplementary file 1 [file jmb-31-4-529-supple.pdf]

J. Microbiol. Biotechnol.
https://doi.org/10.4014/jmb.2012.12045

Table S1. Primers used in this study

Primer	Uses	Sequence (5′ - 3′)	Annealing temperature (ºC)	Expected size of PCR product (bp)
SeIfV1	RT-PCR and RT-qPCR	CATTCAAGACGGTTACACCATTC<br>GACTTTGAATACACGGGACGG	52	457
SeIfV2	RT-PCR and RT-qPCR	GAGTCCATCGTTCACCTTGGC<br>TAGGGGAGCCACAGAGGACTTG	52	297
SeMNPV	PCR and qPCR	CCGCTCGCCAACTACATTAC<br>GAATCCGTGTCGCCGTATATC	52	149
T7_SeIfV1[1]	RNAi	TAATACGACTCACTATAGGGAGA CATTCAAGACGGTTACACCATTC<br>TAATACGACTCACTATAGGGAGA GACTTTGAATACACGGGACGG	54	503
T7_SeIfV2	RNAi	TAATACGACTCACTATAGGGAGA GAGTCCATCGTTCACCTTGGC<br>TAATACGACTCACTATAGGGAGA TAGGGGAGCCACAGAGGACTTG	54	343
Recombinant bacteria	RNAi	CATTCAAGACGGTTACACCATTC<br>GGGAACAACATTTACCGGTTCCT	52	188
dsCON	RNAi	CCCACTAGTGTCCTCATCACCTCCTCAAAC<br>CCCAAGCTTCAGAGTCACCGTTGCAAGTA	54	520
RL32	RT-PCR and RT-qPCR	ATGCCCAACATTGGTTACGG<br>TTCGTTCTCCTGGCTGCGGA	52	270

[1] Underlined sequence indicates T7 promoter.
